# Perceptions of Data Set Experts on Important Characteristics of Health Data Sets Ready for Machine Learning: A Qualitative Study

**DOI:** 10.1001/jamanetworkopen.2023.45892

**Published:** 2023-12-01

**Authors:** Madelena Y. Ng, Alaa Youssef, Adam S. Miner, Daniela Sarellano, Jin Long, David B. Larson, Tina Hernandez-Boussard, Curtis P. Langlotz

**Affiliations:** 1 Department of Medicine (Biomedical Informatics), Stanford University School of Medicine, Stanford, California; 2 Department of Biomedical Data Science, Stanford University School of Medicine, Stanford, California; 3 Department of Radiology, Stanford University School of Medicine, Stanford, California; 4 Department of Psychiatry and Behavioral Sciences, Stanford University School of Medicine, Stanford, California; 5 Department of Pediatrics, Stanford University School of Medicine, Stanford, California

## Abstract

**Importance:**

The lack of data quality frameworks to guide the development of artificial intelligence (AI)-ready data sets limits their usefulness for machine learning (ML) research in health care and hinders the diagnostic excellence of developed clinical AI applications for patient care.

**Objective:**

To discern what constitutes high-quality and useful data sets for health and biomedical ML research purposes according to subject matter experts.

**Design, Setting, and Participants:**

This qualitative study interviewed data set experts, particularly those who are creators and ML researchers. Semistructured interviews were conducted in English and remotely through a secure video conferencing platform between August 23, 2022, and January 5, 2023. A total of 93 experts were invited to participate. Twenty experts were enrolled and interviewed. Using purposive sampling, experts were affiliated with a diverse representation of 16 health data sets/databases across organizational sectors. Content analysis was used to evaluate survey information and thematic analysis was used to analyze interview data.

**Main Outcomes and Measures:**

Data set experts’ perceptions on what makes data sets AI ready.

**Results:**

Participants included 20 data set experts (11 [55%] men; mean [SD] age, 42 [11] years), of whom all were health data set creators, and 18 of the 20 were also ML researchers. Themes (3 main and 11 subthemes) were identified and integrated into an AI-readiness framework to show their association within the health data ecosystem. Participants partially determined the AI readiness of data sets using priority appraisal elements of accuracy, completeness, consistency, and fitness. Ethical acquisition and societal impact emerged as appraisal considerations in that participant samples have not been described to date in prior data quality frameworks. Factors that drive creation of high-quality health data sets and mitigate risks associated with data reuse in ML research were also relevant to AI readiness. The state of data availability, data quality standards, documentation, team science, and incentivization were associated with elements of AI readiness and the overall perception of data set usefulness.

**Conclusions and Relevance:**

In this qualitative study of data set experts, participants contributed to the development of a grounded framework for AI data set quality. Data set AI readiness required the concerted appraisal of many elements and the balancing of transparency and ethical reflection against pragmatic constraints. The movement toward more reliable, relevant, and ethical AI and ML applications for patient care will inevitably require strategic updates to data set creation practices.

## Key Points

**Question:**

What makes data sets for artificial intelligence (AI) ready for health and biomedical machine learning (ML) research purposes?

**Findings:**

In this qualitative study consisting of interviews with 20 data set experts who are creators and/or ML researchers, participants largely appraised data set AI readiness with a set of intrinsic and contextual elements, described what they perceived as optimal characteristics of AI-ready data sets, and provided insights on what factors facilitate the creation of AI-ready data sets. Ethical acquisition and societal impact emerged as appraisal considerations that have not been described in prior data quality frameworks.

**Meaning:**

The findings of this study suggest that strategic updates to data set creation practices are warranted in the advent of AI and ML to better develop reliable, relevant, and ethical clinical applications for patient care.

## Teaser

This qualitative study examines the perceptions of data set experts on the present status of development of artificial intelligence (AI)–ready data sets for use in machine learning research.

## Introduction

Clinical artificial intelligence (AI) applications have the potential to improve patient care and advance biomedical research. Machine learning (ML) research is already producing AI models across a spectrum of disease areas.^1,2,3^ Central to ML research is the data from which models are trained. To accelerate ML discoveries and meet an ethical obligation to treat health data as a public good,^4^ many health data sets have been publicly released to support growing calls for open science and transparency in ML research.^5,6,7^ However, ML models derived from these data continue to be criticized for lacking usefulness, reliability, and fairness.^8,9^

Many of these challenges are inextricably attributed to the quality of data sets. Making data sets AI ready or high-quality and useful for the development of ML applications in health care is often an intensive process that requires coordination across the data preparation pipeline.^10^ Most available data sets lack diversity^11,12^ and have a paucity of high-quality labels necessary for ML, including diagnoses, demographic characteristics, and other critical elements of clinical context. Consequently, only a small fraction of open health data sets (eg, COVID-19–related data sets) contain the clinically relevant annotations to support generalizable ML research.^13,14^

Machine learning models reflect the episodic decisions of stakeholders across the AI life cycle.^15^ The proper use and reuse of AI-ready data sets by researchers is also integral to preventing harmful bias in ML models used for patient care and resource allocation.^16,17,18^ Therefore, producing unbiased AI-ready data sets requires a comprehensive understanding of these issues to combat the dynamic unpredictability of ML model development.

A definition of what constitutes AI-ready data sets for ML remains elusive. We drew on existing data quality frameworks as a guiding tool for our evaluation. Despite numerous frameworks with established data quality dimensions,^19^ including those specific to big data,^20,21,22,23,24,25,26,27,28^ ethics,^29^ and ML,^30^ none fully integrate the nuances required for ML research in health care or considerations that are conducive to AI-ready data set creation in practice. The lack of frameworks to guide the development of AI-ready data sets limits their usefulness for ML research in health care and prevents us from attaining diagnostic excellence.^31^ We envision an AI-readiness framework that is informed by both conventional expectations of data quality and the contemporary needs of ML researchers. Such a framework can lead to greater understanding of how to strengthen data set production and data sharing for clinical AI innovation. In this study, we explored the perspectives of data set creators and ML researchers to determine what makes health data sets AI ready.

## Methods

The Stanford School of Medicine Institutional Review Board reviewed and approved this qualitative study, with a waiver of documentation of consent. Participants provided verbal consent to be interviewed and received an information sheet stating that findings/data may be published in scientific journals. Participants did not receive financial compensation. This study followed the Consolidated Criteria for Reporting Qualitative Research (COREQ) reporting guideline.

### Study Design, Participants, Recruitment

We conducted qualitative interviews of experts involved in the creation of data sets and/or their use for ML research. The semistructured interview gathered participant demographic characteristics, data roles, role responsibilities, and perspectives on data set AI readiness and related topics.

We identified eligible experts who were involved in the creation of publicly available health data sets or the use of these data sets for ML research. Some experts met both criteria. Starting with a list of health data sets or databases, we relied on accessible sources that included consulting respective database web pages, associated publications (scientific or media), collaborators and other experts, and the open web to identify and corroborate eligible experts and obtain contact information. Purposive sampling, or the intentional selection of information-rich individuals,^32^ was used to optimize inclusion of participants from diverse data sets and organizational sectors. From August 23, 2022, to January 5, 2023, we recruited 20 participants after approaching 93 eligible experts with an email invitation; nonrespondents were sent a follow-up email. Race was documented to provide information about participants and potential perspectives that may not have been included.

### Data Collection

Data collection occurred in 2 stages during a scheduled interview session. All interviews were conducted in English through a secure video conferencing platform by the team leader (M.Y.N.). First, participants were asked to verbally complete a survey on demographic characteristics, data roles, and role responsibilities (eMethods in Supplement 1). Second, the interviewer used a semistructured interview guide developed with the study team to gather participant perspectives. Interview questions focused on optimal characteristics of AI-ready data sets and their associated facilitators and barriers. The semistructured format allowed for both focused discussions and probing questions during interviews. Interviews were video and audio recorded and transcribed verbatim. Interviews were conducted until reaching thematic saturation, defined as the point where no new codes or themes emerge from the data.^33^

### Data Analysis

We used quantitative content analysis to categorize and count frequencies of specific content from the survey responses.^34,35^ Thematic analysis^36,37,38^ that drew on techniques of grounded theory^39,40^ was used to identify themes or patterns from the interview data. Interview transcripts were imported into MaxQDA 2022.^41^ First, the team leader (M.Y.N.) generated initial codes from the raw interview data using inductive and deductive approaches. Deductive codes were selected to organize the interview data into broad content areas (eg, optimal characteristics, facilitators, and barriers) during initial coding. An initial codebook was created with both inductive and deductive codes. Second, team members (M.Y.N., A.Y., and D.S.) independently coded, line-by-line, a subset of transcripts with these emergent codes. Disagreements were resolved via discussion until consensus was reached. The initial codebook was iteratively refined throughout the coding process. The team leader (M.Y.N.) reviewed all coded transcripts and applied revisions where appropriate to align with the refined codebook; consensus among team members (M.Y.N., A.Y., and D.S.) was reaffirmed. In addition, identified core concepts and connections between categories were shared among the entire study team to triangulate key themes of data set AI readiness.

### Framework Development

We endeavored to develop a framework that depicts the data set quality elements specific to ML research and relevant connections. Framework development occurred in 2 discrete steps. First, we compiled a list of possible data quality elements to consider deductively, informed by select data quality frameworks (eTable in Supplement 1). Second, once themes were identified, we iteratively refined and organized the most relevant themes to create a data set AI-readiness framework. The study team reviewed and approved the final framework.

## Results

### Characteristics of Participants and Represented Data Sets

A total of 20 experts in data set creation and ML research were interviewed (Table 1). Of these participants, 11 individuals (55%) identified as male and 8 (40%) as female; 15 (75%) were younger than 49 years, with mean (SD) age, 42 (11) years. In terms of race, 6 individuals (30%) identified as Asian, 1 (5%) as multiracial, and 12 (60%) as White. All demographic data were self-reported; 1 (5%) participant did not provide this information. While 18 (90%) participants identified as both data creators and ML researchers, 2 (10%) identified primarily as data set creators. Participants were involved in various tasks across the data preparation pipeline,^10^ with most involved in data curation (90%), data documentation (85%), and data analysis (85%). The mean (SD) duration of the interviews was 49 (11) minutes. Participants worked across diverse data sets and databases, as shown by their general characteristics and select traits relevant for clinical data reuse (eg, repository type, longitudinal observations, and research accessibility) (Table 2).^42^ We identified 3 themes, each with subthemes (Table 3) and corresponding salient quotations (Table 4).

**Table 1.** Participant Characteristics

Characteristic	Participants, No. (%) (N = 20)
Race	
Asian	6 (30)
Multiracial^a^	1 (5)
White	12 (60)
Not reported	1 (5)
Age, y	
20-29	1 (5)
30-39	10 (50)
40-49	4 (20)
50-59	1 (5)
60-69	3 (15)
Not reported	1 (5)
Gender	
Female	8 (40)
Male	11 (55)
Not reported	1 (5)
Data role	
Data set creator	20 (100)
Data set user	20 (100)
Machine learning researcher	18 (90)
Responsibilities in role	
Data acquisition	14 (70)
Data deidentification	12 (60)
Data curation	18 (90)
Data storage	16 (80)
Data annotation	15 (75)
Data documentation	17 (85)
Data analysis	17 (85)
Obtain IRB approval	15 (75)
Data set sharing/dissemination	17 (85)
Data set management	16 (80)

Abbreviation: IRB, institutional review board.

^a^ Multiracial includes those who identify as mixed race or selecting more than 1 race.

**Table 2.** Characteristics of Participant-Affiliated Data Sets and Databases

Affiliated data set/database	Data source	No. of sources	Repository type	Longitudinal observations	Research accessibility	Data type^a^	Organization
1000 Genomes	Collaborative (consortium)	Multiple	Collection	No	Open	G	Academic, government
All of Us Research Program	Collaborative (data linkage and harmonization)	Multiple	Registry	Yes	Open, with restrictions	E, G, I, O	Government
CHERISH	The Medical City	Single	Study	No	Not publicly available	E, I, O	Academic^b^
ClinGen/ClinVar	Collaborative (consortium)	Multiple	Collection	No	Open	G	Government
Diverse Dermatology Images	Stanford Medical Center	Single	Study	No	Open, with restrictions	I	Academic
ENCODE	Collaborative (consortium)	Multiple	Collection	No	Open	G	Academic, government
fastMRI	NYU Langone Health	Single	Study	No	Open, with restrictions	I	Academic
gnomAD	Collaborative (consortium)	Multiple	Collection	No	Open	G	Academic, government
Google Research	Varies per data set	Multiple	Collection	Yes	Open, with restrictions	E, I, O	Industry/private
Health Data Research UK	Varies per data set	Multiple	Collection	Yes	Open, with restrictions	E, G, I, O	Government, nonprofit^b^
MIMIC	Beth Israel Deaconess Medical Center	Single	Warehouse	No	Open, with restrictions	E	Academic
National COVID Cohort Collaborative	Collaborative (data linkage and harmonization)	Multiple	Collection	Yes	Open, with restrictions	E	Government
Nightingale Open Science	Varies per data set	Multiple	Collection	No	Open, with restrictions	E, I	Nonprofit
RadFusion	Stanford Medical Center	Single	Study	No	Open, with restrictions	E, I	Academic
Stanford Knee MRI with Multi-Task Evaluation	Stanford Medical Center	Single	Study	No	Open, with restrictions	I	Academic
UK Biobank	Collaborative (data linkage and harmonization)	Multiple	Registry	Yes	Open, with restrictions	E, G, I, O	Government, nonprofit^b^

^a^ Data type categories: E, electronic health record; G, genomic; I, medical imaging; O, other (eg, sensor and laboratory data).

^b^ Created outside of the US.

**Table 3.** Themes and Subthemes

Theme	Description
**Theme 1: Intrinsic elements of data set AI readiness**	
Subtheme	
1	Accuracy
2	Completeness
3	Consistency
4	Ethical acquisition
**Theme 2: Contextual elements of data set AI readiness**	
Subtheme	
5	Fitness
6	Societal impact
**Theme 3: Drivers of AI-ready data sets**	
Subtheme	
7	Data availability
8	Data quality standards
9	Documentation
10	Team science
11	Incentivization

Abbreviation: AI, artificial intelligence.

**Table 4.** Illustrative Quotations Organized by Theme and Subtheme

Theme and subtheme	Identifier	Illustrative quotation
**Intrinsic elements of data set AI readiness**		
Accuracy	1.1	“Extremely clear labels. Here are all these labels of all these outcomes that we’re interested in, here is exactly how this decision was made, here are all the images that are neatly linked to this EHR data, and all of the EHR data is in these very clean joinable tables, and there are no missing values. Every column is declared with the correct type, it is within this date range, and you can check that. It’s very easy to perform a histogram.”
Completeness	1.2	“Depending on the [research] question,…the ideal data set [for machine learning] has all the potentially valuable inputs at really high granularity and really low missingness, and in highly reliable formats where there doesn’t appear to be a lot of misclassification and miscoding….”
Consistency	1.3	“There are artifactual differences between data sets….[When] data sets are generated consistently…with exactly the same experimental parameters, the same assays, the same reagents, the same lab….If all those variables can be fixed, that makes machine learning much easier.”
Ethical acquisition	1.4	“For this particular data set, that’s not possible [to link EHR data] just because these are samples from 40 years back, so not everybody’s going to be alive to provide all of that, and also it wasn’t the intention.…You would have to go with the fresh, new consent and look who wanted to be approached.…If this wasn’t done at the study design level and then done prospectively, then it becomes a challenge.”
	1.5	“I’ve run [1 research study] where none of [the consent forms on data sharing] existed when we did these scans, but these scans are really important for the current development of machine learning techniques to look for cardiovascular disease.…To be able to use these historic consented cohorts and research, I’ve had to go back to the research ethics committee…so they can approve the use of nonconsented imaging.…[The team] worked very hard…with the ethics committee to put together all of these safety controls and authorizations, and a whole package of work around that so that researchers can apply to use the [data set] without having to get extra ethics…approval for every single project.”
**Contextual elements of data set AI readiness**		
Fitness	2.1	“There’s the intended context, so defining when you are building your model, how do you imagine it would be used, and what is the population you’re interested in? How is the population you’re interested in different from the population that’s represented in the data set?…What you need to do is think about the mismatches between these things, like how is what you have [in the data set] different from what you want to have.”
	2.2	“Whatever the real-world variety is, whether that’s sizes of patients, whether that’s types of scanners, image quality, different representative sexes, races, a variety of disease.”
Societal impact	2.3	“Making sure that you’re not encouraging bad papers, bad studies to take place. If someone submitted a data set and I thought that the labels would encourage some kind of unethical research, like someone submitted some data that was pictures of bodies, pictures of faces, and sexuality, and I thought the risk the machine learning community would pick this up and start trying to develop algorithms that would classify someone’s sexuality based on a photo of their face, then I would think, well, I don’t think that’s ethical research, and personally I’m not going to be involved in sharing a data set that encourages that kind of research.”
**Drivers of AI-ready data sets**		
Data availability	3.1	“The best thing for a data set is just lots of people looking at it and using it because that’s how you find biases. That’s how you find errors. You can do that a bit with algorithms, but it is no replacement yet for just having lots of invested people interrogate a data set.”
	3.2	“One thing that’s important is that data sets shouldn’t be treated as siloed. I think oftentimes what happens is that there’s maybe 4 or 5 data sets that are out there, and they’re treated independently…but they’re really part of this consortium of data and being able to quickly identify where data is sourced from, how we can put different sources of data together, I think is very important.”
	3.3	“One of the key blockers of doing really state-of-the-art medical research is that there are no public gimongous data sets that people can use or analyze to train really state-of-the-art machine learning stuff. It all has to take place siloed at the institution level. You really get limited by what’s available at your specific university….It creates this almost data equity question where some universities with really talented people just can’t play in the same game because the data is locked. There’s just no way to get that scale of data.”
Data quality standards	3.4	“Very early on in [data set creation], [team member] made a point about the FAIR principles and…made sure we had APIs and that outside users can access [the data set]….A lot of people were more concerned about the work of [the data set] and us getting our work done…and a little less of a focus on how are we going to share this with the world.”
	3.5	“There are all these decisions that really matter for machine learning…[that] aren’t really systematically captured…Standards matter, so putting things in standard formats, that’s really not been very successful. There’s just a lot of data out there [but] no one’s paid down this debt of bringing it up to comply with all the things we’ve discovered or invented in the past couple years.…[The data] is there but not quite usable in the way you’d hope….”
Documentation	3.6	“It’s always critical to be open about where the data came from and how it was sourced, and what is the data really talking about? It doesn’t necessarily mean that it’s good or bad if it’s smaller or larger, more representative or not of different kinds of populations. I think it’s just important to be clear about it because different data sets can be used in different cases.”
	3.7	“A lot of people coming to us have a lot of machine learning experience… [but] they don’t necessarily have the particular experience of using this type of data. For this particular data type, there are tools out there for them to use, so how can we help them get familiar with those tools so that they can then utilize this particular data….That’s where some of this documentation… has been most useful.”
	3.8	“An actual fully fledged online documentation, which is organic and able to grow because what ends up happening…is you can’t think of all the different research questions that people might try to answer.”
Team science	3.9	“I think the most important step is to establish a team of trustworthy people, well trained, with experience…meaning it’s not just clinicians, it’s also epidemiologists…computer scientists…data scientists…AI experts, model creation experts, machine learning experts who meet regularly.”
	3.10	“A data set is created by human beings….The world we live in now, or the world where data sets function best, or are of most help, is populated by not just people who gather data…[but] by other stakeholders…most importantly patients.”
	3.11	“Setting up data for machine learning is so often an exercise in deriving variables, simplifying the raw data, and decreasing dimensionality, it is critical that whoever is doing that work has a deep understanding of how the data were collected to begin with, so that when they are simplifying things, they are not totally losing the thread of reality because otherwise, you [or your model] can make some pretty crazy conclusions.”
Incentivization	3.12	“I feel like in many ways I’ve kind of destroyed my career by spending too much time on cleaning data and sharing data. Genuinely, I think career-wise, it’s not a good thing to do.…People don’t see [data sets] as research, so there’s a question of are you actually doing research….There’s a lot of work involved in supporting data sets, and academia doesn’t reward data sharing.”
	3.13	“Most of the other hospitals would rather sell the data than make it available for research, and a lot of them actually do. So, you have the challenge of hospitals being money-making institutions in America, which is an incentive that is driving a different use of the data, not an altruistic one, but also the problem that it can be difficult to convince someone to take on non-zero risk in distributing patient data so that the group benefits… I think that’s what stands in the way of really broad, multicenter, rich data sets.”

Abbreviations: AI, artificial intelligence; APIs, application programming interface; EHR, electronic health record; FAIR, findability, accessibility, interoperability, and reusability.

### Intrinsic Elements of Data Set AI Readiness

Inherent characteristics of AI readiness that are independent of ML use case include accuracy, completeness, consistency, and ethical acquisition. These categories are most relevant to the reliability dimension of data quality, which is defined as whether a user can trust the data.^23^

#### Accuracy

Participants expressed that accuracy consists of well-defined labels as well as ground truth annotations for training and testing of ML models. Participants emphasized the importance of having labels and annotations that are good measurements of what the model intends to predict (Table 4, quotation 1.1). Thus, the accuracy of labels and annotations are core to AI readiness. Documentation that provides supportive proof or describes how labels and annotations were generated can further enhance AI readiness.

#### Completeness

Completeness or meeting an expectation of comprehensiveness contributes to AI readiness. Characteristics such as the size, granularity, breadth, diversity, low missingness, and temporality of data provide indications of data set completeness (Table 4, quotation 1.2). Data sets are considered more AI ready when they contain a comprehensive picture of the area of study (eg, patient journey). Larger data sets are hence preferred for ML research because they increase the likelihood that a desired level of data set completeness will be attained.

#### Consistency

Consistency in data creation, acquisition, and preprocessing is an important expectation. Artificial intelligence readiness is more likely when data are generated using equivalent methods, variables are collected and coded in a similar manner, and the data are harmonized to the intended use (Table 4, quotation 1.3).

#### Ethical Acquisition

Ethically acquired data are also fundamental to AI readiness. A major determinant of ethical acquisition is whether informed consent was obtained from data contributors that allows for broad and originally unintended secondary use of the data. Data sets without proper permissions should not be used by ML researchers. Data sets that rectify informed consent deficiencies across its data sources are inherently more ready for ML research as it is less likely that research endeavors and integrity will be compromised (Table 4, quotation 1.4-1.5).

### Contextual Elements of Data Set AI Readiness

Contextual characteristics of AI readiness that depend on the ML use case include fitness and societal impact. Fitness is pertinent to the relevance dimension of data quality.^23^ Societal impact is aligned with the ethical dimension of data quality, which explores the ethical implications of the use of subpar data sets.

#### Fitness

Participants described characteristics of fitness, or whether a data set meets the requirements of a particular ML research task. Each biomedical ML research task has a unique set of requirements that dictate data set fitness. Users determine a data set’s fitness for use for ML research by assessing the alignment between the ML task requirements and the data set contents. Data set fitness requires appraisal of contextual information across the life cycle of the ML task, including its intended purpose, the target population compared with the populations represented in the data set, and the eventual deployment environment (Table 4, quotation 2.1)

Representativeness of the data helps users appraise data set fitness for an ML research task. Participants noted the importance that the target population in which the ML model will be deployed is represented in the data set. The heterogeneity of a data set can be measured not only on sociodemographic factor and health outcomes, but also on the diversity of health care sites, resource settings, expertise levels, and geographic locations (Table 4, quotation 2.2).

#### Societal Impact

When determining AI readiness, participants considered the societal implications of data set use. Users feel an obligation to assess the risks, harms, or biases that may arise. Machine learning tasks or models developed for health or biomedical purposes have unique ethical, societal, and safety implications that differentially impact populations, which may be further exacerbated through the use of inappropriate or imbalanced data sets (Table 4, quotation 2.3).

### Drivers of AI-Ready Data Sets

Participants divulged contributors that affect user appraisals of data set AI readiness. These contributors include the state of data availability, data quality standards, documentation, team science, and incentivization.

#### Data Availability

Participants were supportive of making health data sets publicly available and considered it beneficial to AI readiness. Open access to data sets enables users to appraise AI readiness and identify areas for improvement. Data set shortcomings are more easily discovered through actual use and public scrutiny (Table 4, quotation 3.1). The recognition that data sets are not independent can further enhance AI readiness. Although a data set on its own may not be ready for a particular ML research task, it may achieve a sufficient level of AI readiness when combined with other data sets (Table 4, quotation 3.2). However, systemic inequities in data availability continue to hinder the creation of AI-ready data sets, which ultimately limit the potential of ML research (Table 4, quotation 3.3).

#### Data Quality Standards

Data quality standards and frameworks contribute to AI readiness, but their application and use appear to be highly variable. Some teams were adamant about incorporating data quality standards during data set creation while others chose to optimize data quality elements or dimensions in an ad hoc manner. Nevertheless, one participant attributed the prolonged usefulness of their data set to the incorporation of FAIR (findability, accessibility, interoperability, and reusability) data principles,^43^ a set of guidelines that help enhance the reusability of data sets (Table 4, quotation 3.4). Data quality standards and frameworks need to be updated to fully drive AI readiness. Current standards do not adequately convey what matters most in ML research or how data set creators can bring a host of data set debt into compliance (Table 4, quotation 3.5).

#### Documentation

Participants cited documentation as a key contributor to AI readiness. The extent of documentation, especially qualitative contextual information about data provenance and data processing decisions, helps users make decisive judgments about AI readiness for their specific ML research tasks. Important accompanying documentation includes data origination, data collection circumstances, data contributor sociodemographic characteristics, intended uses, data preprocessing decisions, label and annotation generation decisions, recommended tools and resources, and other information necessary for robust and fair model development. The transparency of this information clarifies a data set’s purpose and caveats, so that users can inspect, select, and use the data set they deem most appropriate (Table 4, quotation 3.6). It also indicates the limitations that may arise in developing the ML model. Furthermore, data sets with comprehensive documentation shorten the learning curve for ML researchers and allow for efficient data use (Table 4, quotation 3.7). Documentation also needs to be up-to-date and living to account for changes to a data set after its publication date and the evolving research landscape (Table 4, quotation 3.8).

#### Team Science

Participants mentioned the benefits of team science as another driver of AI readiness. The makeup of a team, including diversity of expertise, training, and experiences, contributes to more comprehensive and thoughtful construction of data sets (Table 4, quotation 3.9). Informed teams can yield operational decisions that enhance intrinsic and contextual elements of AI readiness. Furthermore, well-formed teams from reputable institutions can enhance the perceived trustworthiness of the data sets produced. Patients and other data contributors should be considered as part of an effective data set creation team (Table 4, quotation 3.10). A component of AI readiness is whether the data set contains relevant information about the needs and health outcomes of the populations they aim to serve. Keeping knowledgeable humans in the loop throughout data set creation is also considered essential for maintaining AI-ready data sets (Table 4, quotation 3.11).

#### Incentivization

The professional incentivization of data set work would support the creation of AI-ready data sets. Those involved in data set creation and quality maintenance have noted the increasing labor required to meet the latest demands of researchers, yet resources and funding for that work remain lacking. Professionals in academia, as expressed by one respondent, are less inclined to be invested in data quality work as the system largely rewards those who use the data for research (Table 4, quotation 3.12). Some participants also noted the limits of their individual effort in creating and sustaining quality data sets, since they are subject to the constraints of organizational decisions, motivations, and risk tolerance (Table 4, quotation 3.13). Incentivization (ie, direct benefits to funding, resources, or reputation) also needs to be aligned with the organization to compel systemic changes that facilitate AI-ready data set creation.

### Framework for Data Set AI Readiness

We mapped these themes onto a framework to show their association within the health data ecosystem (Figure). The framework consists of 3 core components: (1) drivers of AI-ready data sets, (2) elements of data set AI readiness, and (3) the health data ecosystem.

**Figure.**
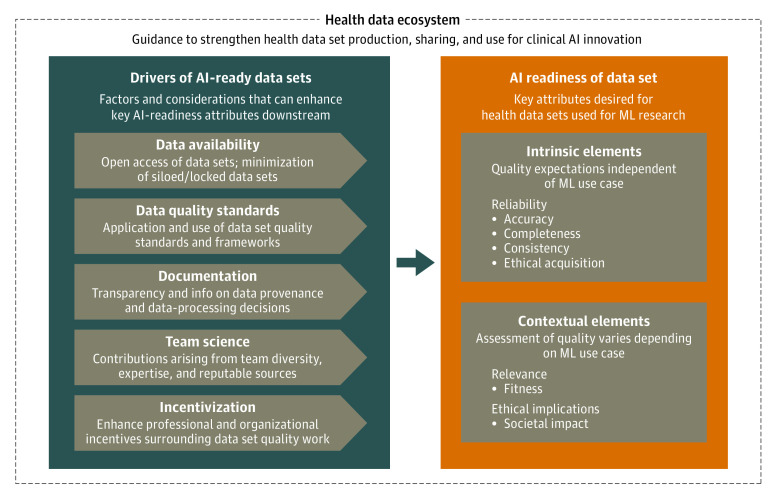
Framework for Measuring the Artificial Intelligence (AI) Readiness of Health Data Sets for Machine Learning (ML) Research This AI-readiness framework serves as a guide for data set stakeholders to consider when creating health data sets for ML research. The framework incorporates data quality expectations and provides context about the contemporary needs of ML researchers.

## Discussion

Our study set out to delineate what constitutes an AI-ready data set that is useful for ML research in health and does not perpetuate harm and bias. We sought perspectives from experts working with a broad spectrum of data sets. Using themes grounded in their perspectives, we developed a broadly applicable AI-readiness framework that informs data set stakeholders about the most relevant data set quality metrics for ML research and considerations to recapitulate a facilitating environment for AI-ready data set creation.

We strived to distinguish how our framework varied from existing data quality frameworks. Accuracy, completeness, consistency, and fitness were entrenched expectations and have been well described across many data quality frameworks. Machine learning researchers partially determined the AI readiness of data sets using these priority appraisal characteristics. Ethical acquisition and societal impact emerged as expectations of our participant sample that have not been described in prior frameworks. Ethical considerations permeated AI-readiness discussions, reflecting a key challenge in the ML research landscape. This increased emphasis is likely due to recent ethical controversies in ML research, including the misuse of user data, privacy and data breaches, and cases of algorithmic bias.^16,17,18^ Our framework recognizes how they appraise not only the historical ethical aspects of a data set (ie, permissions allowed by original informed consent and data use agreements) but also the prospective ethical impact of data set use (ie, production of fair algorithms).

Our framework also identifies factors that drive creation of high-value health data sets and mitigate risks associated with data reuse, which may negatively affect patient care decisions, limit research potential, and waste important resources. These driving factors affect elements of AI readiness and hence ML researchers’ overall perception of data set usefulness. There are several factors that drive AI readiness. The first is availability, which aligns with the community’s call to make data sets open access or easily accessible, thereby increasing collaboration and reproducibility.^6^ Open access data sets are subject to continuous public auditing, which can surface hidden biases. Despite these advantages, there remains resistance to open data set sharing.^44^

Data quality standards can provide a systematic guide to data set creation and drive AI readiness. Standards are a set of aspirational recommendations that can help address common shortcomings in most contexts (eg, using a common data model). The advantages of data quality standards must be balanced by the practicality of adhering to standards in lower resourced health care settings. Data from these sources may not be considered AI ready according to some data quality standards, but their inclusion would nonetheless be valuable for addressing the biases and imbalances in data sets. These ethical trade-offs are important to consider before enforcement of data quality standards.

Documentation drives AI readiness by enhancing the transparency of the data set creation process. Documentation provides stakeholders with known data set quality information,^45^ so that they may decide whether a data set meets the threshold of AI readiness sufficient for their research needs. Improving documentation for health data sets, such as with the addition of datasheets,^46^ healthsheets,^47^ or data set nutrition label^48^ provides ML researchers with key information necessary to facilitate decision-making with model development and meet downstream model reporting guidelines.^9,49^ Comprehensive documentation encourages an equitable ecosystem in which diverse ML researchers can more easily access, understand, and use health data sets appropriately.

Team science is another driver of AI readiness. Team science recognizes the value of diverse cross-disciplinary teams for helping solve multifaceted problems.^50,51^ Diverse and inclusive AI teams are integral to bias mitigation,^52^ with compounded benefits if implemented at the data set creation stage. For example, jury learning, in which diverse annotators make data labeling decisions, meaningfully altered classification outcomes.^53^ Diverse teams can create more relevant and informed labels, further contributing to data set AI readiness.

In addition, the value of incentives to drive AI readiness points to the need for more resources invested in the data set quality workforce. Data set creation and maintenance are underappreciated by traditional metrics of academic productivity. Data set creators made decisions that impact AI readiness (eg, deciding to not carry out clinical annotations) due to the lack of resources and incentives for such tasks. While data set creators may feel a moral obligation to continue high-touch maintenance and oversight after the public sharing of a data set, they often provide this service to the detriment of their professional growth. Given the appreciating value of quality health data sets for ML research, incentives, and resources need to be aligned (eg, National Institutes of Health funding initiatives and journal requirements) for those involved in data set quality work to meet AI-readiness metrics, manage developing risks, and be recognized for their contributions.

### Limitations

Our study was limited by the sample size. Responses from the 20 participants may not be representative of all data set experts. Data set use and requirements for AI and ML research are also rapidly evolving and in flux. Thus, our work represents a snapshot in time. Future work will require frequent updates to data quality frameworks and the meaning of AI readiness.

## Conclusions

The AI readiness of health data sets is a key factor in clinical AI and ML innovation. This qualitative study developed a grounded framework for AI data set quality. Our work suggests that the concept of data set AI readiness is complex and requires the concerted appraisal of many elements and the balancing of transparency and ethical reflection against pragmatic constraints. The movement toward more reliable, relevant, and ethical ML research will inevitably require strategic updates to data set creation practices.

## Notes

Supplement 1. **eMethods.** Survey on Participant Demographics, Data Roles, and Role Responsibilities **eTable.** Data Quality Dimensions, Elements, and Attributes for AI-Readiness Framework Development

Supplement 2.
Data Sharing Statement
