# Supplementary material for: Perceptions of Data Set Experts on Important Characteristics of Health Data Sets Ready for Machine Learning: A Qualitative Study
Source: JAMA Netw Open. 2023 Dec 1;6(12):e2345892. doi: 10.1001/jamanetworkopen.2023.45892 (PMC10692863; doi:10.1001/jamanetworkopen.2023.45892)
Supplement: Supplement 1. — eMethods. Survey on Participant Demographics, Data Roles, and Role Responsibilities eTable. Data Quality Dimensions, Elements, and Attributes for AI-Readiness Framework Development [file jamanetwopen-e2345892-s001.pdf]

Supplementary Online Content

Ng MY, Youssef A, Miner AS, et al. Perceptions of data set experts on important characteristics of health data sets ready for machine learning. *JAMA Netw Open*. 2023;6(12):e2345892. doi:10.1001/jamanetworkopen.2023.45892

eMethods. Survey on Participant Demographics, Data Roles, and Role Responsibilities

eTable. Data Quality Dimensions, Elements, and Attributes for AI-Readiness Framework Development

This supplementary material has been provided by the authors to give readers additional information about their work.

© 2023 Ng MY et al. *JAMA Network Open.*

eMethods. Survey on Participant Demographics, Data Roles, and Role Responsibilities

Demographic Questions

- Race/ethnicity
- Age
- Sex
- State
- Country

Current role

Which of the following positions best describes your current role? (please check all that apply)

- ☐ **Ethics Officer:** Defines and develops ethical standards that support safe and responsible data-sharing at organization.
- ☐ **Compliance/Regulatory Officer:** Oversees ethical and legal compliance within the organization, and ensures compliance with laws, regulatory requirements, policies, and data management procedures.
- ☐ **Privacy Officer**: Evaluates organization health data de-identification policies and procedures for research in compliance with HIPAA.
- ☐ **Dataset Creator:** Person responsible for the creation of the dataset used for AI health applications.
- ☐ **Machine Learning Researcher:** Person responsible for the use of the dataset for the development of AI models for health applications.

Sub-roles

Given your position, what specific role did you play in the public data creation/sharing/dissemination process?

- ☐ **Data acquisition** - played a role in consolidation self-reported, EHR, sensors/wearable, genetic data, or other data relevant to the dataset
- ☐ **Data de-identification** - played a role in the data de-identification process
- ☐ **Data curation** - played a role in aggregating, selecting, organizing, harmonization, and managing data to meet the needs of researchers or other stakeholders
- ☐ **Data storage** - played a role in identifying an appropriate storage space for the data
- ☐ **Data annotation** - played a role in the modification of the data to contain unique research information
- ☐ **Data documentation** - played a role in process of recording any aspect of project design, sampling, data collection, cleaning and analysis that may affect results
- ☐ **Data analysis/usage to create AI models** - played a role in using the dataset data to create AI models for healthcare applications
- ☐ **Obtain IRB approval to use and share the dataset** - played a role in obtaining institutional approval (e.g., IRB) to use and share the dataset
- ☐ **Data-sharing authorization (organization-level)** - authorizes the public release of health data for machine learning (ML) or artificial intelligence (AI) research and/or application development
- ☐ **Sharing/dissemination of the dataset (individual-level)** - played a role in sharing/disseminating the dataset
- ☐ **Dataset management (individual-level)** - played a role in the continuous management of the dataset
- ☐ **Dataset management (organization-level)** - monitors the management of clinical databases for AI research and applications development across the organization

eTable. Data Quality Dimensions, Elements, and Attributes for AI-Readiness Framework Development

Data Quality Dimensions, Elements, Attributes	Definitions *(Compiled and summarized, where applicable)*	Reference[a,b]										
		19	21	22	23	24	25	26	27	28	29	30
Accessibility, access	Data are available, easily and quickly retrievable; difficulty level for users to obtain data; linked to data openness.<br>● Dimensional clusters: (i) accessibility, access security;[19] (ii) accessibility, availability;[20] (iii) access, security.[29]	D,A	D,A		A					D,A		
Accuracy	Data are correct, reliable, and certified free of error; data recorded correctly and reflects realistic values; accuracy of a given data value compared to a known reference value; extent to which data accurately represents the real world.<br>● Dimensional clusters: (i) accuracy, correctness, validity, and precision.[20]	A	D,A	A	A	A	A	A	A	A		*
Appropriate amount of data, quantity, volume	The extent to which the quantity or volume of available data is appropriate.	A						A		A		*
Auditability	Auditors can fairly evaluate data accuracy and integrity within rational time and manpower limits during the data use phase.				A							
Authorization	Whether an individual or organization has the right to use the data.				A							
Availability	The degree of convenience for users to obtain data and related information.<br>● Dimensional clusters: (i) accessibility, timeliness, authorization.[22]		A		D							
Believability	The extent to which data are accepted and regarded as true, real, and credible.	A	A							A		
Completeness	The extent to which data are of sufficient breadth, depth, and scope for the task at hand. Describes whether all relevant data, and all its components, are recorded. Can be measured by missing/incomplete values and records.<br>● Dimensional clusters: (i) completeness, pertinence, relevance.[20]	A	D,A	A	A	A		A	A	A		*
Comprehensiveness	Dataset contains all representative samples from the population. Also include clarity and simplicity of data.		A									A

© 2023 Ng MY et al. *JAMA Network Open.*

Conciseness, concise representation	The extent to which data are compactly represented without being overwhelming (i.e., brief in presentation, yet complete and to the point). Also include minimality and compactness of data.	A	A							A		
Confidentiality	To achieve data integrity and informed policy making based on accurate and valid data. Determines whether the right data is in the right hands and is secure.						A					
Consistency	Data agrees with its format and structure; logical relationship between correlated data is correct and complete. In a database, it means whether the same data located in different storage areas should be considered equivalent (i.e., the data have equal value and the same meaning or are essentially the same). Consistency ensures internal validity. The capability of data to comply without contradictions to all properties of the reality of interest. Also includes cohesion and coherence of data.<br>● Dimensional clusters: (i) consistency, cohesion, coherence.[20]		D,A	A	A	A	A	A	A	A		*
Contextual	Data must be considered within the context of the task at hand.<br>● Dimensional clusters: (i) value-added, relevance, timeliness, completeness, appropriate amount of data;[19] (ii) believability, relevance, value-added, quantity, accessibility, reputation.[29]	D				*				D		
Correctness	A record in a dataset is accurate and valid, and they are correctly labeled if they are labeled records.		A			*						A
Credibility	Refers to the objective and subjective components of the believability of a source or message. Credibility of data has three key factors: reliability of data sources, data normalization, and the time when the data are produced. Indicates how meaningful and credible the data is.				A				A			
Data protection	Concerns the ways to secure data, algorithms and models against unauthorized access.										A	
Definition/documentation	Data specification, which includes data name, definition, ranges of valid values, standard formats, etc.				A							
Distinctness	The absence of duplicates and measures the percentage of unique registrations or distinct attribute values in a dataset.							A				
Diversity	The degree to which different kinds of objects are represented in a dataset.										A	
Ease of understanding	The extent to which data are clear without ambiguity and easily comprehended.	A	*							A		

© 2023 Ng MY et al. *JAMA Network Open.*

Ethics	Data should conform to a high ethical standard; ethical implications of its usage.<br>● Dimensional clusters: (i) fairness, transparency, diversity, data protection.[27]										D	
Fairness	The lack of bias.										A	
Fit for purposes, fitness	Important to the performance of machine learning. Fitness has two-level requirements: (1) the amount of accessed data used by users and (2) the degree to which the data produced matches users' needs in the aspects of indicator definition, elements, classification, etc.<br>● Dimensional clusters: (i) comprehensiveness, correctness, variety.[28]				A							D
Integrity	In a database, data integrity means having a complete structure. Data values are standardized according to a data model and/or data type. In information security, data integrity means the data cannot be modified in an unauthorized or undetected manner.				A							
Interpretability	The extent to which data are in appropriate language and units and the data definitions are clear.	A								A		
Intrinsic	Data have quality in their own right.<br>● Dimensional clusters: (i) believability, accuracy, objectivity, reputation;[19] (ii) completeness, consistency, accuracy, timeliness.[29]	D				*				D		
Manipulability	Related to 'representational' dimension; refers to the extent the data can be organized or altered for ease of understanding.									A		
Meta data	Meta data describes different aspects of the datasets to reduce the problems caused by misunderstanding or inconsistencies.				A							
Objectivity	The extent to which data are unbiased (unprejudiced) and impartial.	A										
Pedigree/lineage	Helps in knowing the source of the data so that any inconsistency is corrected in the source and not in any other instances.			A								
Pertinence	Related to representing the relevant aspects of the reality of interest.<br>● Dimensional clusters: (i) consistency, credibility, freshness.[26]		A						D			
Precision	Granularity of data; the degree with which the values of an attribute are close to each other.		A	A				A				

© 2023 Ng MY et al. *JAMA Network Open.*

Presentation Quality	A valid description method for the data, which allows users to fully understand the data.<br>● Dimensional clusters: (i) readability, structure.[22]				D							
Readability	The ability of data content to be correctly explained according to known or well defined terms, attributes, units, codes, abbreviations, or other information.<br>● Dimensional clusters: (i) readability, comprehensibility, clarity, simplicity.[20]		D,A		A							
Redundancy	The capability of representing the reality of interest with the minimal use of informative resources.<br>● Dimensional clusters: (i) redundancy, minimality, compactness, conciseness.[20]		D,A									
Relevance, relevancy	The degree of correlation between data content and users' expectations or demands. The extent to which data are applicable and helpful for the task at hand.<br>● Dimensional clusters: (i) fitness.[22]	A	A	A	D					A		
Reliability	Whether we can trust the data.<br>● Dimensional clusters: (i) accuracy, integrity, consistency, completeness, auditability;[22] (ii) accuracy, completeness, uniqueness.[26]				D				D			
Representational	Importance of the role of systems in presenting data (e.g, same format, compatible with previous data).<br>● Dimensional clusters: (i) interpretability, ease of understanding, representational consistency, concise representation;[19] (ii) interpretability, manipulability, ease of understanding, conciseness of representation, representational consistency.[29]	D,A								D,A		
Reputation	The extent to which data are trusted or highly regarded in terms of their source or content; judgment made by a user to determine the integrity of a source.	A	A							A		
Security, access security	The extent to which access to data can be restricted and hence kept secure.	A								A		
Structure	Level of difficulty in transforming semi-structured or unstructured data to structured data through technology.				A							

© 2023 Ng MY et al. *JAMA Network Open.*

Timeliness, freshness	Age of the data is appropriate for the task at hand; data is up to date; meaningful analysis within time delay from data generation and acquisition to utilization; data are temporally valid; describes the degree to which the data is current for specific needs.	A		A	A	A		A	A	A		*
Transparency	The ability to interpret the information extraction process in order to verify which aspects of the data determine its results. Transparency metrics can include data provenance and explanation.										A	
Trust	How much data derives from an authoritative source, irrespective of the ethical implications of use.<br>● Dimensional clusters: (i) trust, believability, verifiability, reputation.[20]		D,A				*					
Uniqueness	New records have to be unique when compared to other datasets. There is only one entry of its kind.								A			
Usability	Whether the data are useful and meet users' needs.<br>● Dimensional clusters: (i) definition/documentation, credibility, meta data;[22] (ii) transformation, conformity, storage penalty, normalization, referential integrity.[26]				D				D			
Validity	Conformance of data values to a domain.		A									*
Value-added	The extent to which data are beneficial and provide advantages from their use.	A								A		
Variety	The coverage of a dataset of all different cases on selected features.											A
Verifiability	The degree by which a data consumer can assess the correctness of the data set.		A									

[a]Data quality categories: D, dimension, usually contains a cluster of attributes; A, attribute, standalone quality element as denoted by reference; and *, potentially relevant quality element discussed alongside the reference's explicit framework.
[b]Reference 20 is a systematic literature review and excluded.

© 2023 Ng MY et al. *JAMA Network Open.*
