# Supplementary material for: Perceptions of Data Set Experts on Important Characteristics of Health Data Sets Ready for Machine Learning: A Qualitative Study
Source: JAMA Netw Open. 2023 Dec 1;6(12):e2345892. doi: 10.1001/jamanetworkopen.2023.45892 (PMC10692863; doi:10.1001/jamanetworkopen.2023.45892)
Supplement: Supplement 2. — Data Sharing Statement [file jamanetwopen-e2345892-s002.pdf]

Data Sharing Statement

Ng. Perceptions of Data Set Experts on Important Characteristics of Health Data Sets Ready for Machine Learning. *JAMA Netw Open*. Published December 01, 2023. doi:10.1001/jamanetworkopen.2023.45892

Data
Data available: Yes
Data types: Other (please specify)
Additional Information: Codebook
How to access data: madelena@stanford.edu
When available: With publication

Supporting Documents
Document types: None

Additional Information
Who can access the data: Researchers who would like to review the codebook.
Types of analyses: N/A
Mechanisms of data availability: With investigator support.
